# Prevalence, antibiotic resistance pattern for bacteriuria from patients with urinary tract infections

**DOI:** 10.1002/hsr2.2039

**Published:** 2024-04-11

**Authors:** Md. Jubayer Hossain, Abul Kalam Azad, Md. Shahadat Bin Shahid, Muhibullah Shahjahan, Jannatul Ferdous

**Affiliations:** ^1^ Population Health Studies Division, Center for Health Innovation, Research Action, and Learning – Bangladesh (CHIRAL Bangladesh) Dhaka Bangladesh; ^2^ Department of Microbiology Jagannath University Dhaka Bangladesh; ^3^ Department of Transfusion Medicine Mugdha Medical College and Hospital Dhaka Bangladesh

**Keywords:** antibiotic resistance, Bangladesh, susceptibility, urinary tract infections (UTI), uropathogen

## Abstract

**Background and Aims:**

Antibiotic resistance presents a significant global public health challenge, particularly for urinary tract infections (UTIs), and is notably severe in developing countries. Surveillance of the antimicrobial susceptibility patterns of UTI‐causing bacteria is crucial for effective treatment selection. This study aimed to analyze these patterns in bacteria isolated from the urine samples of patients at Mughda Medical College Hospital, Dhaka, Bangladesh.

**Methods:**

A retrospective study (January 2019 to December 2020) at Mugdha Medical College and Hospital, Dhaka, examined clinical and laboratory data from patients with positive urine cultures (≥10^5^ CFU/mL). The study classified patients into four age groups: children (1–<18 years), young adults (18–<33 years), middle‐aged adults (33–50 years), and old adults (>50 years). The standard Kirby‐Bauer method was used to assess antibiotic sensitivity to 28 common antibiotics.

**Results:**

Among 243 positive urine cultures in both community‐ and hospital‐acquired UTIs, *Escherichia coli* was the most common uropathogen (65.84%), followed by *Klebsiella* spp. (12.34%), *Enterococcus* spp. (8.23%), and other types of bacteria.

**Conclusion:**

Old adults are particularly vulnerable to UTIs, with *E. coli* being the predominant causative agent in the study region. The observed antimicrobial resistance patterns underscore the necessity of judicious antibiotic selection to effectively treat UTIs across different age groups.

## 1 INTRODUCTION

Urinary tract infection (UTI) is the second most common infection, affecting 150 million people, both males and females, annually worldwide.^1^, ^2^, ^3^, ^4^, ^5^ However, females are more susceptible to UTIs than males because their urethra is typically shorter and more conveniently located near the rectum, which makes them vulnerable to uropathogenic bacteria and causes infection.^3^, ^6^, ^7^, ^8^, ^9^, ^10^, ^11^, ^12^ More than 77% of UTI patients are females of various ages.^13^, ^14^, ^15^ A previous study showed that sexually active women are more susceptible to UTIs than men.^16^ One in five women may have a UTI at some point in their life. Repeated UTIs are more likely to occur if someone has already experienced UTIs. In addition, 27% of women experience two or more episodes annually.^12^, ^17^, ^18^, ^19^ Owing to less concern regarding hygiene, old adults (>65 years) had the highest incidence, followed by young adults.^20^ Therefore, UTIs complicate the daily lives of people of all ages, notably middle‐aged adult women and the old‐adult population.

Pain or burning during urination, frequent urination, bloody urine, feeling the need to urinate despite having an empty bladder, and pressure or cramping in the groin or lower abdomen are some of the consequences of UTI. Therefore, these symptoms complicate the livelihoods of the affected individuals, and the treatment of UTIs is urgent. Antibiotics are commonly used to treat UTIs, because they are often caused by bacterial agents. However, fungi and viruses can also cause UTIs.^21^, ^22^, ^23^, ^24^ Taking antimicrobial drugs only by observing the symptoms of UTIs rather than by proper laboratory diagnosis is one of the main reasons for the evolution of resistant organisms. Self‐medication and overuse of antimicrobial drugs are practiced by many people in Bangladesh [18–20]. Consequently, the long‐term misuse of antimicrobial agents has increased antibiotic resistance, which has become a chronic health concern.^25^, ^26^, ^27^, ^28^ The long‐term use of antimicrobials can eradicate the growth of normal urethral flora, thus maximizing the growth of pathogenic bacteria.^26^, ^28^, ^29^ Antimicrobial resistance of microbes is a rigorously evolving phenomenon. It is crucial to maintain a continuous surveillance regime to monitor susceptibility patterns to different commonly used antibiotics. Developmental nations such as Bangladesh do not adhere to the World Health Organization's recommendations in this regard, whereas developed nations do.

In a previous study, 920 (29%) of 4500 samples from Bangladesh were UTI‐positive.^6^, ^30^ In another study, prevalence was highest in the summer.^31^, ^32^ In more than 60% of cases, microorganisms exhibit resistance to commonly used antibiotics, such as Cefixime, Ciprofloxacin, Amoxicillin, Nalidixic acid, and Cephalosporins.^33^ These facts raise ineligibility difficulties associated with UTI treatment. Detailed profiling of individual pathogens is necessary. The study population comprised a wide range of ages (1–95), which were divided into different age groups, thus providing a thorough proportional understanding of vulnerability patterns of UTI infection in different age groups.^34^ In addition, only reports of infected individuals were collected deliberately, which ensured a focused antibiotic susceptibility analysis with minimal bias.

This study aimed to investigate the recent status of prevalence of bacteriuria infection and its antimicrobial susceptibility patterns among UTI‐positive patients at Mugdha Medical College Hospital. This will also help to understand the antimicrobial resistance pattern and choose appropriate antibiotics to treat UTI.

## 2 METHODS

### 2.1 Study design and population

This retrospective study was conducted at the Mugdha Medical College and Hospital in Dhaka, spanning a period of 2 years from January 2019 to December 2020. This study aimed to analyze the clinical and laboratory findings of individuals exhibiting positive urine cultures with a bacterial count of 10^5^ colony‐forming units per mL (CFU/mL) of urine. The patient cohort was stratified into four distinct age groups: children and adolescents (1–<18 years), young adults (18–<33 years), middle‐aged adults (33–50 years), and old adults (>50 years). Subsequently, an antibiotic sensitivity assay was conducted using the widely recognized Kirby‐Bauer method to assess the susceptibility of the isolated bacteria to the 28 most frequently prescribed antibiotics.

### 2.2 Data collection tools

Data were collected from laboratory reports that contained information about uropathogenic bacterial isolates, bacterial sensitivity, and antibiotic resistance.

### 2.3 Data collection

This was a retrospective study conducted by collecting information from medical files, such as the date of sample collection, sex and age of patients, bacterial isolates, antibiotics prescribed, and concomitant diseases.

### 2.4 Statistical analysis

Data were entered into a database designed using a Google Sheet and analyzed using MS Excel (version 2013) software. Retrospective study statistics showed the percentage of uropathogens and antibiotic resistance/sensitivity over a particular period. The overall 2‐years resistance/sensitivity rates of the most common uropathogens to routinely tested first‐line antimicrobials were calculated by dividing the number of urinary isolates that were resistant or sensitive to each antimicrobial agent by the number of isolates tested against an individual antimicrobial agent. Tables are presented as frequencies (counts) and percentages.

## 3 RESULT

### 3.1 Distribution of UTI among gender and age groups of the patients

A total of 243 UTI‐positive samples were selected for this study, which consisted of 200 (82%) female and 43 (18%) male patients, indicating that the prevalence of UTI in female patients was much higher than that in male patients. Patients were divided into four age groups to track the distribution of uropathogens according to age. The highest (34%) prevalence of UTI was found among old adults (>50 years), followed by young adults (24%), middle‐aged adults (23%), and children and adolescents (19%) (Table 1).

**Table 1:** The distribution of UTI among gender and age groups of patients.

Name of isolates (number of isolates)	Sex		Age groups			
	Female	Male	1–<18	18–<33	33–50	>50
*E. coli* (160)	139 (87%)	21 (13%)	29 (18%)	41 (26%)	39 (24%)	51 (32%)
*Klebsiella* (30)	22 (73%)	8 (27%)	5 (17%)	6 (20%)	6 (20%)	13 (43%)
*Enterococcus* (20)	17 (85%)	3 (15%)	4 (20%)	7 (35%)	2 (10%)	7 (35%)
*Streptococcus* (16)	14 (88%)	2 (12%)	0 (0%)	4 (25%)	6 (38%)	6 (38%)
*Proteus* (7)	2 (29%)	5 (71%)	6 (86%)	0 (0%)	1 (14%)	0 (0%)
*Pseudomonas* (6)	3 (50%)	3 (50%)	1 (17%)	0 (0%)	0 (0%)	5 (83%)
*Staphylococcus* (4)	3 (75%)	1 (25%)	1 (25%)	1 (25%)	1 (25%)	1 (25%)
Total	200 (82%)	43 (18%)	46 (19%)	59 (24%)	55 (23%)	83 (34%)

Abbreviation: UTI, urinary tract infection.

### 3.2 Distribution of UTI‐causing bacteria

In this study, among the identified 243 uropathogens, 212 (87%) were monomicrobial growth and 31 (13%) were polymicrobial growth. Detailed analysis showed that uropathogenic *Escherichia coli* (UPEC) and *E. coli* were the most prevalent bacteria (65.85%), followed by *Klebsiella* spp. (12.35%) and *Enterococcus* spp. (8.23%), *Streptococcus* spp. (6.58%), *and Proteus* spp. (2.88%) and *Pseudomonas* spp. (2.47%) and *Staphylococcus* spp. (1.64%), respectively (Table 2).

**Table 2:** Distribution of UTI‐causing bacteria.

Bacterial isolates	Frequency (%)
*E. coli*	160 (65.85)
*Klebsiella*	30 (12.35)
*Enterococcus*	20 (8.23)
*Streptococcus*	16 (6.58)
*Proteus*	7 (2.88)
*Pseudomonas*	6 (2.47)
*Staphylococcus*	4 (1.64)

Abbreviation: UTI, urinary tract infection.

### 3.3 Distribution of uropathogens among different gender and age groups

Furthermore, we analyzed the distribution of uropathogenic bacteria among different sexes and age groups. The prevalence of *Streptococcus* spp., *E. coli*, *Enterococcus* spp., *Staphylococcus* spp., and *Klebsiella* spp. was higher in females than in males. In contrast, the prevalence of *Proteus* spp. was higher in male patients compared to in women. A similar prevalence of *Pseudomonas* spp. was observed in both sexes.

In distribution among age groups, results of the study showed that *E*. *coli* was more prevalent in old adults (32%) and less prevalent in children and adolescents (18%). A gradual increase in the age groups of young and middle‐aged adults has been observed.

Similar results were obtained for *Klebsiella* spp., with the highest prevalence in old adult patients (43%) and the lowest prevalence in children and adolescents (17%). In the case of young and middle‐aged adults, the prevalence rates of these isolates were quite similar.

In the case of *Enterococcus* spp., the results showed the highest prevalence in both young adults (35%) and old adults (35%), followed by moderate (20%) in children and adolescents, and a low prevalence in middle‐aged adults (10%).

The highest prevalence of *Streptococcus* spp. was observed in middle‐aged adults and old adults (38%), whereas in young adult patients, the rate was moderate (25%). However, the prevalence rate was not found in children and adolescents.

Surprisingly, *Proteus* spp. were the most prevalent in children (86%), and no cases accounted for the young and old adult age groups.

On the other hand, *Pseudomonas* spp. showed the reverse prevalence of *Proteus* spp.; the highest and the lowest prevalence was highlighted in the old adult age group (83%) and children (17%), respectively. The prevalence of *Staphylococcus* spp. (25%) was equal across all age groups (Table 1).

### 3.4 Antibiotic sensitivity pattern of isolated organism

The antibiotic sensitivity patterns of the identified bacterial isolates showed that *E. coli* had 100%, 86%, 75%, and 62% resistance to Amoxicillin and Fusidic acid, Azithromycin, Ticarcillin, and Nalidixic acid, respectively (Table 3). In contrast, Amikacin, Colistin, Doripenem, Imipenem, Linezolid, Meropenem, Tigecycline, and Vancomycin were effective against *E. coli*, with >90% sensitivity. The other tested antibiotics were moderately effective against *E. coli* (Table 4).

**Table 3:** Proportion (%) of common urinary pathogens showing resistance (R) to antimicrobial agents.

Bacterial isolates	*E. coli*	*Klebsiella*	*Enterococcus*	*Proteus*	*Pseudomonas*	*Staphylococcus*	*Streptococcus*
Amoxicillin	100	0	5.3	0	0	0	0
Azithromycin	86	93	74	100	100	100	50
Amoxyclav	30	40	0	43	100	0	0
Amikacin	3.8	20	76	14	33	0	100
Cefepime	34	37	60	0	67	0	0
Cefotaxime	38	33	62	0	50	25	0
Carbapenem	42	40	79	14	100	75	0
Cloxacillin	0	0	0	0	0	33	0
Ceftriaxone	42	40	79	14	100	75	0
Colistin	1.3	3.3	100	86	0	0	0
Cefixime	51	41	90	17	100	100	12
Ceftazidime	37	37	86	14	17	0	0
Cefuroxime	46	40	65	14	100	0	0
Ciprofloxacin	36	33	65	43	50	50	19
Cefradine	0	0	80	0	100	0	14
Trimethoprim/sulfamethoxazole	38	43	95	57	100	50	94
Clindamycin	0	0	87	0	0	50	27
Doxycycline	38	37	84	71	100	25	50
Doripenem	5.6	20	0	14	17	0	0
Fusidic Acid	100	0	100	0	0	75	50
Gentamicin	14	27	20	14	50	0	75
Imipenem	5.7	17	100	14	40	0	0
Levofloxacin	35	34	55	29	50	50	19
Meropenem	6.2	17	30	14	33	0	0
Nalidixic acid	62	33	100	71	100	0	0
Norfloxacine	0	0	100	0	100	0	0
Nitrofuran	13	82	5.9	86	0	0	0
Piperacillin	11	27	0	14	17	0	0
Tigecycline	1.3	6.7	0	0	67	0	0
Ticarcillin	75	0	100	100	0	0	0

**Table 4:** Proportion (%) of common urinary pathogens demonstrating sensitivity (S) to antimicrobial agents.

Bacterial isolates	*E. coli*	*Klebsiella*	*Enterococcus*	*Proteus*	*Pseudomonas*	*Staphylococcus*	*Streptococcus*
Amoxicillin	0	0	95	0	0	0	100
Ampicillin	0	0	0	0	0	0	100
Azithromycin	4.8	0	11	0	0	0	50
Amoxyclav	58	53	100	57	0	100	100
Amikacin	96	80	18	86	67	100	0
Cefepime	65	63	40	100	33	100	100
Cefotaxime	62	67	38	0	0	50	100
Carbapenem	58	60	16	86	0	0	94
Colistin	99	97	0	14	100	0	0
Ceftriaxone	58	60	16	86	0	0	94
Cefixime	47	59	10	83	0	0	81
Ceftazidime	61	63	14	86	83	0	100
Cefuroxime	53	60	35	71	0	100	100
Ciprofloxacin	45	63	10	43	50	25	25
Cefradine	0	100	13	0	0	100	86
Trimethoprim/sulfamethoxazole	62	57	5.3	43	0	50	6.2
Clindamycin	0	0	13	0	0	50	73
Doripenem	94	80	100	86	83	0	100
Gentamicin	86	73	80	86	50	100	25
Imipenem	94	83	0	86	60	0	0
Levofloxacin	63	66	40	71	50	50	62
Linezolid	100	0	100	0	0	100	100
Meropenem	93	83	70	86	67	100	100
Nalidixic acid	36	67	0	29	0	0	0
Nitrofuran	87	18	94	14	0	100	100
Penicillin G	0	0	0	0	0	0	100
Piperacillin	84	70	100	86	83	100	100
Tetracycline	0	0	100	0	0	0	0
Tigecycline	98	90	100	100	33	100	100
Teicoplanin	0	0	100	0	0	100	100
Vancomycin	100	0	100	0	0	100	100

In the present study, *Klebsiella* spp. was the second most predominant uropathogen, which showed resistance against the antibiotics Azithromycin and Nitrofuran at 93% and 82%, respectively (Table 3). Cefradine, Colistin, Tigecycline, and Amikacin showed 100%, 97%, 90%, and 80% sensitivity against *Klebsiella* spp., respectively (Table 4).

*Enterococcus* spp. was the third most prevalent UTI‐causing bacteria, with 100% resistance to Colistin, Fusidic acid, Imipenem, Nalidixic acid, Norfloxacin, and Ticarcillin; 95% to Cotrimoxazole; 90% to Cefixime; 87% to Clindamycin; 86% to Ceftazidime; 84% to Doxycycline; and 80% to Cefadine (Table 3). A clear visualization of MDR (Multidrug‐resistant) *Enterococcus* spp. was found to be resistant to seven antimicrobial class agents at the same time, among which multiple were from the same antibiotic classes. Cephalosporin, Carbapenem. Contrarily, the bacterial isolates were 100% sensitive to Amoxyclav, Doripenem, Linezolid, Piperacillin/Tazobactam/Tazobactam, Tetracycline, Tigecycline, Teicoplanin, and Vancomycin, 95% to Amoxycillin, 94% to Nitrofuran (Table 4).

*Proteus* spp. are resistant to Azithromycin and Ticarcillin. It also showed high resistance (86%) to Colistin and Nitrofuran (Table 3). Cefepime and Tigecycline were the most potent antibiotics against *Proteus* spp. infections, followed by Amikacin, Carbapenem, Ceftazidime, Cefixime, Ceftazidime, Ceftriaxone, Doripenem, Gentamicin, Imipenem, Meropenem, Piperacillin (86% sensitivity), and Cefixime (83% sensitivity) (Table 4).

In this study, *Pseudomonas* spp. exhibited the highest level of resistance (100%) against ten antibiotics namely, Azithromycin, Amoxyclav, Carbapenem, Cefxime, Ceftriaxone, Cefuroxime, Cefradine, Cotrimoxazole, Doxycycline, Nalidixic acid, and Norfloxacin (Table 3). A clear picture of multidrug‐resistant (MDR) *Pseudomonas* spp. was found to be resistant to six antimicrobial class agents at the same time, among which multiple were from the same antibiotic class, for example, Cephalosporin. In contrast, only one antibiotic, Colistin, was found to be completely effective, followed by three antibiotics, Ceftazidime, Doripenem, and Piperacillin (83% sensitivity), against *Pseudomonas* spp. (Table 4).

*Staphylococcus* spp. isolates were fully resistant (100%) to Azithromycin and Cefixime and moderately resistant (75%) to Carbapenem, Ceftriaxone, and Fusidic acid (Table 3). Interestingly, our results showed that most antibiotics were fully effective against *Staphylococcus* spp. (Table 4).

Interestingly, most antibiotics used in this study, other than Amikacin and Cotrimoxazole, were resistant to *Streptococcus* spp. (Table 3). Ciprofloxacin, a widely used antibiotic, was found to have a high level of intermediacy (56%) in this study (Table 5).

**Table 5:** Proportion (%) of common urinary pathogens exhibiting intermediate (I) response to antimicrobial agents.

Bacterial isolates	*E. coli*	*Klebsiella*	*Enterococcus*	*Proteus*	*Pseudomonas*	*Staphylococcus*	*Streptococcus*
Azithromycin	9.7	6.9	16	0	0	0	0
Amoxyclav	12	6.7	0	0	0	0	0
Amikacin	0	0	5.9	0	0	0	0
Cefepime	1.3	0	0	0	0	0	0
Cefotaxime	0	0	0	0	50	25	0
Carbapenem	0	0	5.3	0	0	25	6.2
Cefixime	1.9	0	0	0	0	0	6.2
Ceftazidime	2.5	0	0	0	0	0	0
Cefuroxime	0.6	0	0	14	0	0	0
Ciprofloxacin	18	3.3	25	14	0	25	56
Cefradine	0	0	6.7	0	0	0	0
Doxycycline	0.6	0	0	0	0	0	0
Levofloxacin	1.3	0	5	0	0	0	19
Meropenem	0.6	0	0	0	0	0	0
Nalidixic acid	2.5	0	0	0	0	0	0
Piperacillin	4.4	3.3	0	0	0	0	0
Tigecycline	0.6	3.3	0	0	0	0	0

## 4 DISCUSSION

This study investigated uropathogenic bacteria that cause UTIs, such as *E. coli*, *Klebsiella* spp., *Enterococcus* spp., *Streptococcus* spp., *Proteus* spp., *Pseudomonas* spp., and *Staphylococcus* spp. *E. coli* was the most prevalent pathogen, followed by *Klebsiella* spp. (12.35%) and *Enterococcus* spp. (*8.23%*), respectively. The most important finding of this study was that the prevalence of *E. coli*, *Klebsiella* spp., and *Pseudomonas* spp. was very high in the old adult group, and most of this uropathogen showed >90% resistance to 10 commonly used antibiotics. Another important finding was that *Proteus* spp. was the most prevalent uropathogen in children and adolescents and was predominant in males. *Enterococcus* spp. showed a higher prevalence in both young and old adults, and *Proteus* spp. was the most prevalent in children. *Staphylococcus* spp. showed equal prevalence among all age groups. Multidrug resistance has been observed in some cases, with varying levels of sensitivity to different antibiotic classes.

The prevalence of UTIs in old adults increases with an increase in the age of individuals aged ≥60 years.^35^, ^36^, ^37^ It has been reported that it increases by almost 30% in women over the age of 85. In fact, the prevalence of UTI in both men and women over the age of 85 years increases significantly.^38^, ^39^, ^40^, ^41^ The result of the study stating that the prevalence of UTIs in the old adult group was higher than that in other age groups is consistent with these findings. The risk factors for high UTI in the aging population were found to be different from those in the younger population.^42^, ^43^, ^44^ The main risk factors were age‐associated changes in immune function, the use of urinary catheters, and the presence of comorbidities such as cancer, diabetes, neurological disorders, and stroke. Prior experience with UTIs, nosocomial infections, obstruction of urine flow, high retention of urine compromising renal and kidney defense mechanisms, presence of prostatic hypertrophy in men, loss of estrogen in postmenopausal women, and certain medications, such as anticholinergic drugs.^45^, ^46^ All of these risk factors make old adults susceptible to UTI.

UTIs are the most common cause of bacteremia in both community‐dwelling and institutionalized old adults with respiratory infections.^47^, ^48^, ^49^, ^50^, ^51^, ^52^, ^53^ Overutilization of antibiotics is generally used to prevent the prevalence of UTIs in old adults.^52^ Consequently, a higher rate of antibiotic‐resistant uropathogens was found in patients after taking oral antibiotics.^10^, ^54^, ^55^ Interestingly, in this study, the highest number of antibiotics were found to be resistant to *Pseudomonas* spp., which was most prevalent in the old adult population. It is necessary to recommend therapeutic techniques other than antibiotics, such as changes in habit and intake of dietary supplements such as cranberry juice drinks, for UTIs that are prevalent in old adult people.^56^

Previous studies have demonstrated that the prevalence of UTIs in female patients is higher than in male patients worldwide, especially in Africa, Asia, and South Asian countries.^10^, ^57^, ^58^ Several studies in Bangladesh have reported similar results.^3^, ^6^, ^7^, ^41^, ^59^ The anatomical structure of women, such as the shorter urethra, being very close to the vagina and anal orifice, hormonal changes during the menopausal period, during sexual intercourse receiving bacteria from their partner, use of contraceptive materials, and involvement in sexual activities using both the vagina and anal orifice, might be the potential reasons for the higher prevalence of UTIs in female patients.^3^, ^60^ In addition, during pregnancy, numerous hormonal and mechanical changes in the body, glycosuria, aminoaciduria, and urine retention in the bladder contribute to a higher prevalence of UTIs in female patients. Thus, females are more susceptible to UTIs than males, as highlighted in the present study data.^58^

Gram‐negative bacteria such as *E. coli, Klebsiella* spp., *Proteus* spp*., and Pseudomonas* spp. are causative uropathogens of UTIs.^10^, ^46^, ^61^ *E. coli* was found as the major causative agent of UTIs in different studies in Bangladesh, India, and all over the world.^3^, ^5^, ^10^, ^62^ It has also been found that *E. coli* is followed by *Klebsiella spp*. as the cause of UTIs.^5^, ^61^ The results of this study are consistent with these studies. Our study also reported the involvement of Gram‐positive bacteria, including *Enterococcus* spp., *Streptococcus* spp., *and Staphylococcu*s spp., in the causation of UTIs, which is supported by previous studies.

The antibiotic resistance patterns of uropathogens vary in their susceptibility to antibiotics based on place and time. Therefore, it is important to routinely analyze the prevalence of uropathogens and their antibiotic susceptibility patterns for appropriate treatment of UTIs. In this study, *E. coli* showed 100% resistance to Amoxicillin, Fusidic acid, 86% to Azithromycin, 75% to Ticarcillin, and 62% to Nalidixic acid. Azithromycin and Nitrofuran were 93% and 82% resistant to *Klebsiella* spp., respectively. *Enterococcus* spp. showed 100% resistance to Colistin, Fusidic acid, Imipenem, Nalidixic acid, Norfloxacin, and Ticarcillin; 95% to Cotrimoxazole; 90% to Cefixime; 87% to Clindamycin; 86% to Ceftazidime; 84% to Doxycycline; and 80% to Cefadine. *Proteus* spp. showed 100% resistance to Azithromycin and 86% to Ticarcillin, Colistin, Nitrofurantoin and 100% sensitivity to Cefepime and Tigecycline. *Pseudomonas* spp. showed 100% resistance to ten antibiotics namely, Azithromycin, Amoxyclav, Cefepime, Ceftriaxone/Rophin, Cefuroxime, Cefradine, Cotrimoxazole, Doxycycline, Nalidixic acid, and Norfloxacin. *Staphylococcus* spp. showed 100% resistance to Azithromycin and Cefixime, followed by moderate resistance (75%) to Ceftriaxone/Rophin, and Fusidic acid. These results indicate that all uropathogens, especially *Pseudomonas* spp. and *Enterococcus* spp., are multidrug‐resistant and affect old adults in the Dhaka South region.^63^ These findings are similar to those of studies conducted in Bangladesh, India, and other developing countries. Some uropathogens also showed differences in susceptibility to antibiotics compared with previous reports. The place and time may explain these differences. Based on these results it could be suggested that physicians should be careful to select antibiotics for these uropathogens causing UTIs patients.

## 5 CONCLUSION

In this study, multiple drug‐resistant (MDR) *Enterococcus* and *Pseudomonas* spp. were found to be completely resistant to most antibiotics from the Cephalosporin class and several of the most frequently used antibiotics. The occurrence of MDR *Enterococcus* spp. infection was higher in females and the prevalence of MDR *Pseudomonas* spp. was higher in the old adult age group and children than in the young and middle‐aged adult age groups. As many commonly used antibiotics are resistant to UTI‐causing bacteria, this study may help to identify effective antibiotics to treat specific UTI agents easily, and performing yearly surveillance systems of antibiotic susceptibility patterns can also make it easier. Antibiotic Stewardship Programs may play a significant role in effectively treating bacterial infections and improving clinical outcomes.

## AUTHOR CONTRIBUTIONS

**Md Jubayer Hossain**: conceptualization; investigation; writing—original draft; writing—review & editing; validation; methodology; software; formal analysis; project administration; data curation; supervision; resources. **Abul Kalam Azad**: writing—original draft; writing—review & editing; supervision; investigation; validation. **Md Shahadat Bin Shahid**: writing—original draft; writing—review & editing; data curation; validation; investigation. **Muhibullah Shahjahan**: writing—original draft; writing—review & editing; data curation; software; investigation. **Jannatul Ferdous**: conceptualization; supervision; writing—original draft; writing—review & editing; data curation; investigation; validation.

## CONFLICT OF INTEREST STATEMENT

The authors declare no conflicts of interest.

## TRANSPARENCY STATEMENT

The lead author Md. Jubayer Hossain affirms that this manuscript is an honest, accurate, and transparent account of the study being reported; that no important aspects of the study have been omitted; and that any discrepancies from the study as planned (and, if relevant, registered) have been explained.

## Data Availability

The datasets used and/or analyzed during the current study are available from the corresponding author upon reasonable request. The data that support the findings of this study are openly available in UTI_Mughda at https://github.com/chiralbd/UTI_Mughda.
